# Al atomistic surface modulation on colloidal gradient quantum dots for high-brightness and stable light-emitting devices

**DOI:** 10.1038/s41598-019-42925-0

**Published:** 2019-04-23

**Authors:** Jae-Sung Lee, Byoung-Ho Kang, Sae-Wan Kim, Jin-Beom Kwon, Ok-Sik Kim, Young Tae Byun, Dae-Hyuk Kwon, Jin-Hyuk Bae, Shin-Won Kang

**Affiliations:** 1 Sensor System Research Center, Korea Institute of Science and Technology (KIST), 5 Hwarang-ro 14-gil, Seongbuk-gu, Seoul 02792 Republic of Korea; 2 Institute of Technology, DONG-A CARBON TECHNOLOGY, 41-3, Gyo 8-Gil, Buksam-eub, Chilgok-gun, Gyeongsangbuk-do Republic of Korea; 3 School of Electronics Engineering, College of IT Engineering, Kyungpook National University, 1370 Sankyuk-dong, Bukgu, 702-701 Daegu, Republic of Korea; 4 Department of Electronic Engineering, Kyungil University, Hayang-up, 712-702 Gyeongsang buk-do Republic of Korea

**Keywords:** Quantum dots, Electrical and electronic engineering

## Abstract

Quantum-dot (QD) light-emitting devices (QLEDs) have been attracting considerable attention owing to the unique properties of process, which can control the emission wavelength by controlling the particle size, narrow emission bandwidth, and high brightness. Although there have been rapid advances in terms of luminance and efficiency improvements, the long-term device stability is limited by the low chemical stability and photostability of the QDs against moisture and air. In this study, we report a simple method, which can for enhance the long-term stability of QLEDs against oxidation by inserting Al into the shells of CdSe/ZnS QDs. The Al coated on the ZnS shell of QDs act as a protective layer with Al_2_O_3_ owing to photo-oxidation, which can prevents the photodegradation of QD with prolonged irradiation and stabilize the device during a long-term operation. The QLEDs fabricated using CdSe/ZnS/Al QDs exhibited a maximum luminance of 57,580 cd/m^2^ and current efficiency of 5.8 cd/A, which are significantly more than 1.6 times greater than that of CdSe/ZnS QDs. Moreover, the lifetimes of the CdSe/ZnS/Al-QD-based QLEDs were significantly improved owing to the self-passivation at the QD surfaces.

## Introduction

Solution-synthesized quantum dots (QDs) have been intensively studied owing to their inherent luminescent characteristics, narrow emission spectral characteristics through particle size control, and high luminescent efficiency with solution processing^1^. The unique properties of the QDs have motivated numerous studies toward their application in next-generation optoelectronic technologies. Since the first directed QD synthesis three decades ago, thin films using QDs have been actively investigated for use in optoelectronic devices such as light-emitting devices (LEDs)^2–4^, solar cells^5^, and photodiodes^6^.

However, QDs suffer from poor photostability and chemical stability against moisture and air, which is still problems their application and commercialization. Furthermore, core-only QDs tend to be unstable in air and provide a low fluorescence quantum yield (QY)^7,8^. Recently, various methods have been developed to address these shortcomings. Several researchers have reported QD stability and improved QY results when growing a shell of a higher-bandgap material in the QD of the core/shell structure^9,10^. Among the shells higher-bandgap, ZnS is widely used for QD passivation, which significantly enhances the stability of the photoluminescence (PL). However, despite the use of shells with higher-bandgap, the originally high QYs are reduced owing to repeated cycles of purification or ligand exchange^11^. A fabrication method of more robust QDs through QD surface protection involves coating of individual QDs with chemically stable metal oxides, which can act as a physical barrier to prevent air and moisture penetration into the QD surface. Many researchers have reported that silica coatings prevent moisture and oxygen from penetrating the QD surface and improve the chemical stability and photostability of QDs^12–15^. However, the formation of silica coatings through the Stöber process is usually accompanied by a considerable QY reduction^16^. In addition, silica-coated QDs are usually powder-type materials and are not well-suited for solution-process-type optoelectronics devices.

Recently, the groups of Yang and Li proposed a method for doping ZnS shells with Al for QD production that is solution processable and effective self-passivation^17,18^. These studies showed that, upon light irradiation, the Al doped in the ZnS shell became photo-oxidized into Al_2_O_3_, which acted as a protective layer and prevented photodegradation of the QDs upon a prolonged irradiation. Although these studies reported a substantial enhancement in the photostability, the complex fabrication process of the individual core and shell, as well as the Al doping, required a long fabrication time. Furthermore, the fabrication of the individual core/shell structure induced a lattice mismatch between the core and shells. Consequently, the highest achievable QY from the as-obtained QDs was smaller than 80%. Therefore, a short and simple synthesis method for QDs, which can improve their photostability and QY, is necessary.

In this study, to improve the QD stability and device performance, we induced Al atomic passivation on the QD surface, and demonstrated its application in a solution-processable QD LED (QLED). To the best of our knowledge, no studies have been reported on self-passivation (e.g., Cd-based core/shell QDs with Al passivated QDs in QLEDs (Al-QLEDs)). Two types of QLEDs were fabricated using CdSe/ZnS and CdSe/ZnS/Al QDs and their electroluminescence (EL) characteristics were compared, revealing significant differences in luminance, efficiency, and lifetime, which reflect the significant impact of the additional Al shell on the device performance. The device with gradient QDs with Al shells exhibited significantly higher luminance, current efficiency, and long-term stability than those of a device with gradient QDs without Al shells. The luminance and current efficiency of the Al QLED are 1.6 times higher than those of the QLED without Al shells. We show that the QLED performance can be modified by the introduction of the Al shells on the QDs in the emissive layer (EML). By analyzing the Al-passivated QDs, we confirmed not only their photostability but also the efficient carrier dynamics and improved lifetime of the Al-QLED. In order to evaluate the performances of the Al-QLEDs, we fabricated solution-processable QLEDs by employing a modification of previously reported methods^19,20^.

## Results

### Structural and optical properties of the QDs

**Figure 1:**
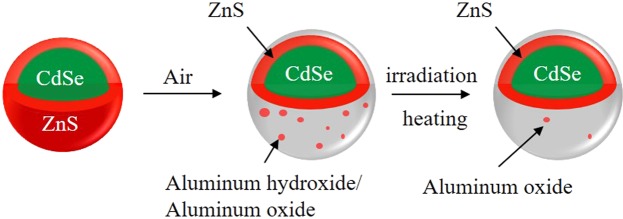
Schematic illustration for transformation process of aluminum oxide shell of QDs.

**Figure 2:**
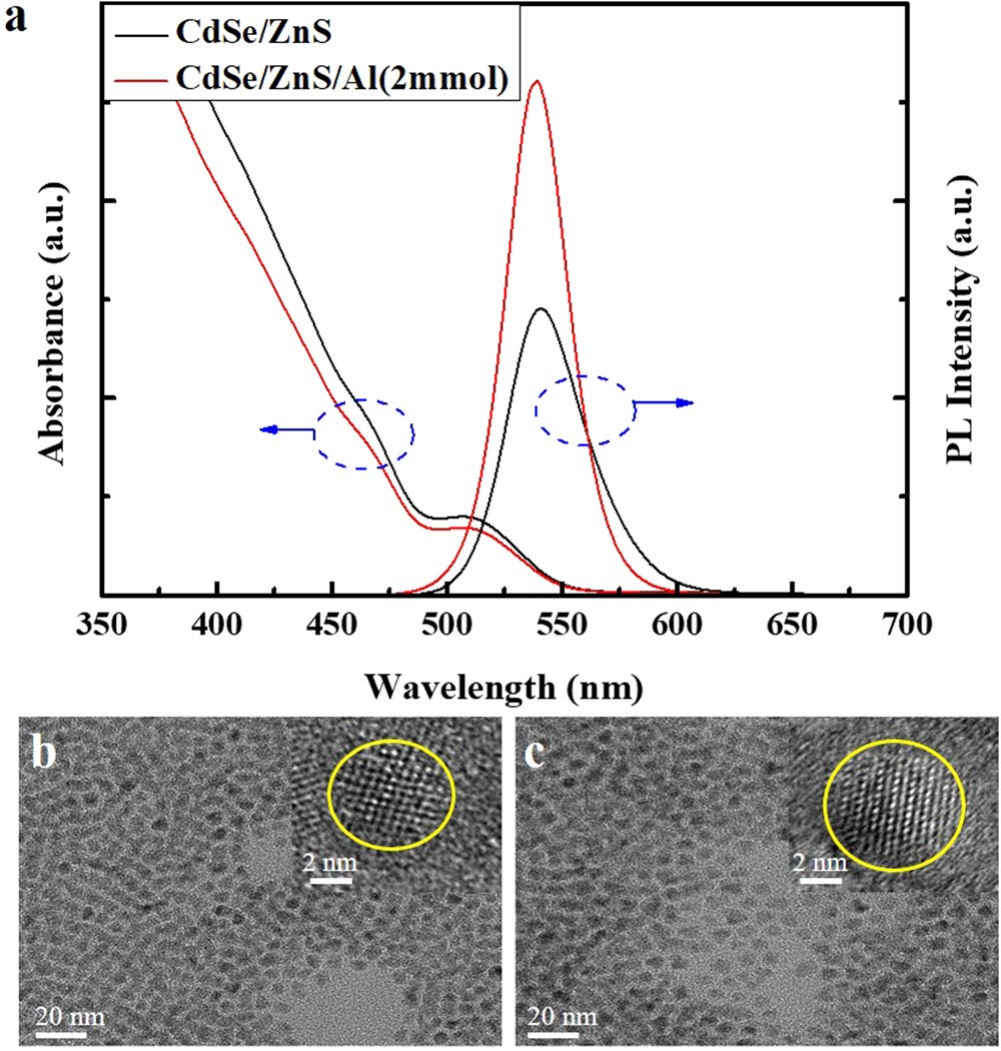
The characteristics of the synthesized QDs. (**a**) Comparison of UV-Vis absorption and PL spectra of CdSe/ZnS and CdSe/ZnS/Al QDs. (**b**,**c**) TEM images (scale bar, 20 nm) of (**b**) CdSe/ZnS QDs and (**c**) CdSe/ZnS/Al QDs with a concentration of 2 mmol.

A simple synthesis method is schematically proposed to phenomenon related to CdSe/ZnS/Al_2_O_3_ QDs in Fig. 1. The Al element was introduced as Al oxide into the shell of ZnS on the CdSe core and then formed a passivation layer when the surface ZnS shell was degraded under under irradiation or heating. The synthesized QD solutions consisted of CdSe/ZnS and CdSe/ZnS/Al QDs with PL peaks at a wavelength of *λ* = 540 nm, as shown in Fig. 2a. It is worth nothing that no significant difference in ultraviolet (UV)-visible absorption spectrum was observed between the QDs with/without the Al shells. These results indicate that there is no significant difference in the energy gap (E_g_) between QDs with and without Al shells. The E_g_ was estimated to be 2.25 eV by converting the original absorption spectrum into a graph of (${ahv}$)^2^ against ${hv}$ (where $a$ = absorbance, $h$ = Planck’s constant, and $v$ = light frequency), and then extrapolating the straight part of the graph to the ${hv}$ axis (Supplementary Fig. S1). The Al shell QDs (CdSe/ZnS/Al) yielded a PL intensity significantly higher than that of the sample without Al. The results indicate that the Al passivation leads to a slight increase in the shell thickness, which can enhance the PL intensity of the QDs by reducing the electron-coupling effect between adjacent QDs. The relative PL QY of the QDs was measured by comparing their PL intensities with those of a primary standard dye solution (Rhodamine 6G) at the same optical density (0.05), at an excitation wavelength of 450 nm^21^. The absolute QY of QD solutions was obtained by absolute PL QY measurement system (OTSUKA Electronics, QE-2000). The PL QY results are similar to the results of the PL intensity. The Al-passivated QDs exhibit a higher PL QY than that of the bare QDs (Supplementary Fig. S2). The highest QY was synthesized with Al overshelling for 2 h; a longer overshelling often worsens the QY (Supplementary Fig. S3). These results show that a more efficient thin-film EML is achieved without Al shelling of the QDs. Figures 1c and 2b show transmission electron microscopy (TEM) images of QDs with/without Al shells, prepared using a coating time of 2 h and doping concentration of 2 mmol. The average sizes of the CdSe/ZnS QDs and CdSe/ZnS/Al QDs were estimated to be 6.7 and 7.6 nm, respectively. Synthesized CdSe/ZnS/Al QDs with Al_2_O_3_ shelling had larger sizes of 0.9 nm-thick Al_2_O_3_. Therefore, the significantly larger average size of the CdSe/ZnS/Al QDs than that of the CdSe/ZnS QDs is entirely attributed to the thicker Al shell.

### Photostability and water stability of QD emitters

**Figure 3:**
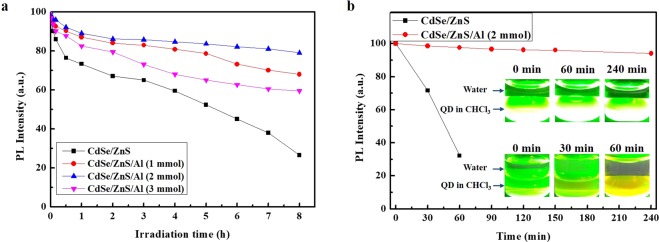
Photostability and water stability analysis of CdSe/ZnS QDs and CdSe/ZnS/Al QDs. (**a**) Effects of the Al doping concentration on the photostability of CdSe/ZnS/Al QDs. (**b**) Water stability of CdSe/ZnS QDs and CdSe/ZnS/Al QDs with a concentration of 2 mmol.

The photostabilities of CdSe/ZnS/Al QDs with different Al doping concentrations are shown in Fig. 3a and compared with that of the normal QDs. The Al shelling of the QDs was performed for 2 h. The QDs had similar sizes to avoid effects of the shell thickness on the photostability. The results indicate that the photostability of the QDs was significantly improved when Al_2_O_3_ was shelled outside the ZnS shell. The PL intensities of all of the QDs decreased with the strong irradiation of light. The QDs without Al_2_O_3_ shelling exhibited rapid PL intensity decays to 50% of the initial PL intensity after 5 h. The Al shelling with an Al doping concentration of 2 mmol yielded the most stable QDs. Their emission was maintained above approximately 80% of the initial intensity for 8 h of operation. A too large Al doping concentration degrades the intrinsic chemical stability of ZnS:Al, because unstable Al-S bonds reduce the photostability of the CdSe/ZnS/Al_2_O_3_ QDs^17,22^. In order to validate the superiority of the moisture barrier of the CdSe/ZnS/Al QDs from a more practical perspective, the QDs were mixed with water under continuous UV irradiation, as shown in Fig. 3b. The PL intensity of the QDs without Al shelling significantly decreased, and the emission peak red-shifted. The PL of QDs without Al_2_O_3_ shells decreased by 68% after 60 min. UV irradiation durations longer than 60 min caused rapid turbidity increases in the QD and water mixture. Compared with the QDs without mixed water, those heated for 60 min exhibited an approximately 8 nm red-shift in the PL, while their absorption peaks were unchanged. On the other hand, the QDs with the Al shells exhibited a good water stability against a prolonged UV irradiation, maintaining 90% of their initial PL even after 2 h of UV irradiation without exhibiting the temporal spectral diffusion. The Al_2_O_3_ shells are very stable in moisture and oxygen, and can act as effective barriers to prevent non-radiative recombination.

### X-ray photoelectron spectroscopy (XPS) and Fourier-transform infrared (FT-IR) analyses

**Figure 4:**
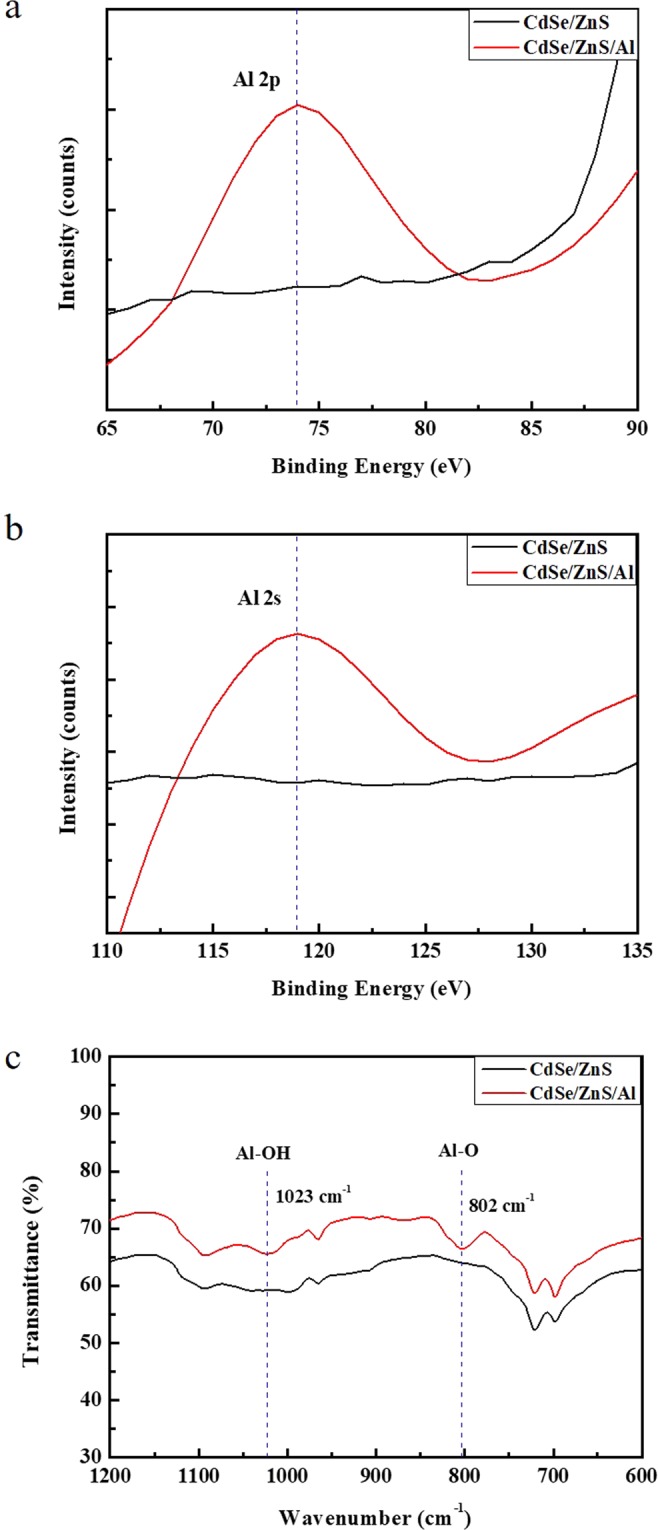
The materials characterization of Al shell on CdSe/ZnS QDs. (**a**–**c**) XPS spectra of the (**a**) Al 2p and (**b**) Al 2 s and (**c**) FT-IR spectra of CdSe/ZnS QDs and CdSe/ZnS/Al QDs with a concentration of 2 mmol.

In order to verify the coordination of the Al_2_O_3_ shell to the CdSe/ZnS QD surface, we analyzed the XPS results and FT-IR spectra of the materials, as shown in Fig. 4. Figure 4,b show typical XPS spectra of the two types of QDs: with and without Al shelling, respectively. After the Al shelling on the CdSe/ZnS QDs, Al 2p and Al 2 s peaks appeared at 74 and 119.3 eV, respectively: these peaks may be associated with oxidized Al species such as Al-OH or Al_2_O_3_ ^23^. As shown in Fig. 4c, an FT-IR spectral peak around 802 cm^−1^ is observed after the Al shelling, related to Al-O vibrations in the Al oxide^22,24^. Additionally, the peak at 1,023 cm^−1^ corresponds to Al-OH in the CdSe/ZnS/Al QDs, which is consistent with the XPS results. Furthermore, we performed energy-dispersive spectroscopy (EDS) (Supplementary Fig. S4) shows the typical EDS of the two types of QDs, with and without Al shelling, along with their compositional results (insets). The Al percentage was calculated to be approximately 4.3%. These results confirm that the Al shell was well prepared on the QD surface.

### Solution-processed QLEDs

**Figure 5:**
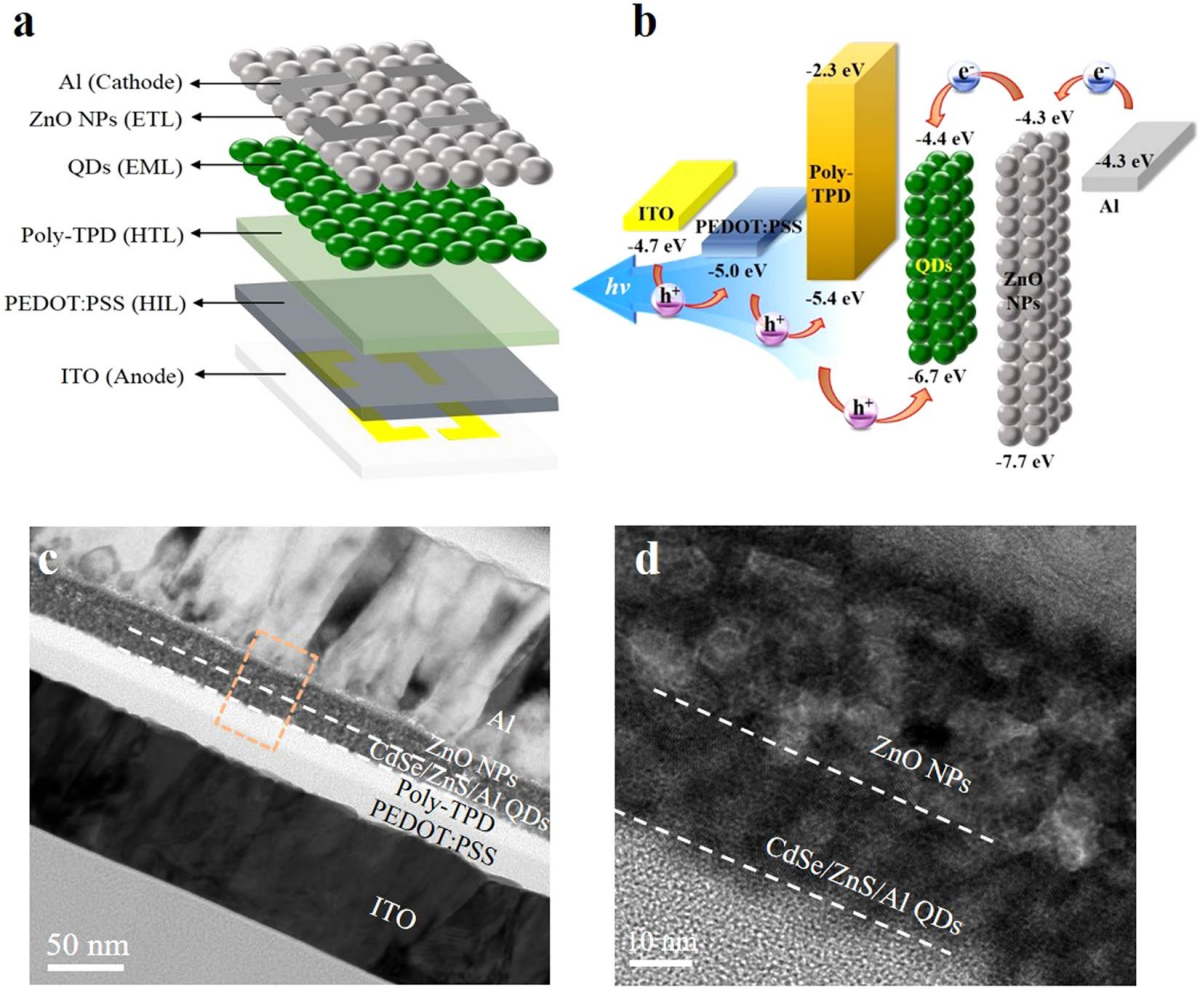
Device structure and TEM images of the solution-processed QLEDs. (**a**) Schematic device structure and (**b**) energy band diagram of the all solution-processed QLED. (**c**) Cross-sectional (scale bar, 50 nm) and (d) higher-magnification (scale bar, 10 nm) TEM images of all solution-processed QLED: ITO//PEDOT:PSS (30 nm)//poly-TPD (45 nm)//CdSe/ZnS/Al QDs (25 nm)//ZnO nanoparticles (30 nm)//Al.

In order to investigate the contribution of the Al shelling of the QDs to the QLED performance, we fabricated QLEDs with three different Al doping concentrations and without Al shelling. Schematics of our device multilayer structures comprising a patterned indium tin oxide (ITO) as the anode, poly(3,4-ethylenedioxythiophene) polystyrene sulfonate (PEDOT:PSS) as the hole-injection layer (HIL), poly(N,N′-bis(4-butylphenyl)-N,N′-bis(phenyl)benzidine (poly-TPD) as the hole-transport layer (HTL), QDs as the EML, ZnO nanoparticles (NPs) as the electron-transport layer (ETL), and Al layer as the cathode, as well as corresponding energy-level diagram and cross-sectional TEM image, are shown in Fig. 5. In order to fabricate the QLEDs, we synthesized two types of QDs with and without Al shelling. Except for the Al cathode, deposited using vacuum thermal evaporation, all of the other layers were sequentially deposited on the ITO by spin-coating. The fabrication of the multilayered structure using the solution process requires the use of orthogonal solvents to ensure the integrity of the underlying layers during the deposition of the overlayers.

### Performances of the QLEDs

The surface ligands of the QDs are important factors affecting the electrical properties of the QLEDs. In general, colloidal synthesized CdSe/ZnS QDs are capped with a long organic ligands. When formed with EML using QDs capped with long organic ligands, these organic surface ligands increase the distance between the individual QDs and exhibit insulating properties that interfere with charge injection into EML. Our as-synthesized CdSe/ZnS QDs and CdSe/ZnS/Al QDs are capped with oleic acid (OA) and 1-dodecanethiol (DDT), respectively. The thiol group capped with QDs (CdSe/ZnS/Al) is much shorter than OA capped with QDs (CdSe/ZnS)^22^. The short ligand length means that there will be reduction interparticle spacing (IPS) of QDs^19,25^. Therefore, reducing the IPS is possible to form a closely-packed EML. The effective confinement of charges from the adjacent layer in the QD EML exhibits a lower turn-on voltage of the QLEDs.

**Figure 6:**
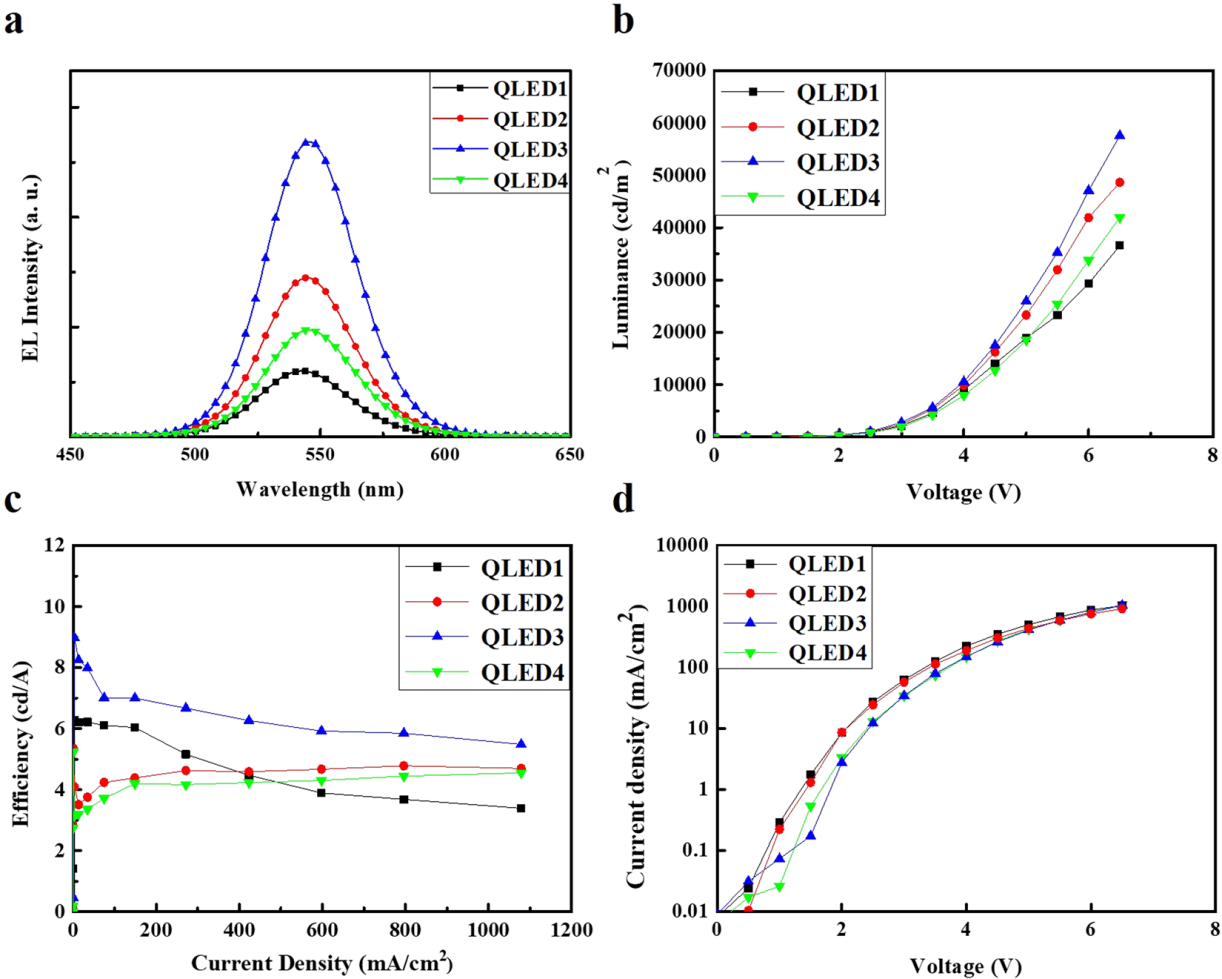
Performance evaluation results for the fabricated QLEDs. (**a**) EL spectra of the QLEDs with an applied voltage of 6 V. Device characteristics of (**b**) voltage-luminance, (**c**) current efficiency-current density, and (**d**) voltage-current density.

The effectiveness of the Al shelling of the QDs was confirmed by measuring their EL spectra, current densities, luminances, and current efficiencies in the QLEDs. Figure 6a shows the performances of the fabricated QLEDs with an applied voltage of 6 V; no parasitic emission is observed from the adjacent layers was observed. The EL intensities of the fabricated QLEDs are approximately 546 nm. Under the same applied voltage, the EL intensity of the sample with Al shelling is significantly higher than that of the sample without Al shelling. The results indicate that the Al shelling can enhance the EL intensity by reducing the number of trap sites.

In order to confirm the contribution of Al shelling of QDs in promoting electron injection and transport, the current densities of electron-only devices with and without Al shelling were measured (Supplementary Fig. S5). The current density of the electron-only device (ITO/Al/QDs(CdSe/ZnS/Al)/Al) is much larger than that of the device (ITO/Al/QDs(CdSe/ZnS)/Al). In the above two devices, the thickness of all layers are identical to those used in the QLEDs. This result clearly demonstrates that electron injection and transport in the devices with CdSe/ZnS/Al QDs are enhanced by the thicker Al shell, which acts as a nontrivial energy barrier against charge injection.

The current densities, luminances, and current efficiencies of the QLEDs with and without Al shelling are presented in Fig. 6b–d, respectively. The results show variations in current density and luminance with a voltage sweep to 6.5 V for four green QLEDs. Four different devices were analyzed as QLED1 (CdSe/ZnS-QD-based QLED), QLED2 (CdSe/ZnS/Al(1 mmol)-QD-based QLED), QLED3 (CdSe/ZnS/Al(2 mmol)-QD-based QLED), and QLED4 (CdSe/ZnS/Al(3 mmol)-QD-based QLED). These devices have low turn-on voltages (≤2.0 V). The maximum luminances of QLED1, QLED2, QLED3, and QLED4 are 36,580, 48,650, 57,580, and 41,940 cd/m^2^, with current efficiencies of 3.39, 4.69, 5.48, and 4.55 cd/A at the maximum luminances, respectively, as summarized in Table 1. These results indicate that QLED3 exhibited the highest performance, which is similar to the case of the photostability. The results of the QDs synthesized through the gradient method indicated that the optimized Al doping concentration is 2 mmol. In addition, they showed that a too large Al concentration worsened the intrinsic chemical stability of ZnS:Al and decreased the device performance owing to the Al–S bonds. The current densities of the CdSe/ZnS/Al-QD-based QLEDs were lower than that of the CdSe/ZnS-QD-based QLED. These results indicated that the shell of CdSe/ZnS/Al QDs thicker than that of CdSe/ZnS QD, indicating a weaker effective electric field and consequently lower charge injection into the EML^21,26^. However, the CdSe/ZnS/Al-QD-based QLED exhibited lower leakage currents and higher luminances characteristics, which was much higher than the CdSe/ZnS-QD-based QLED in terms of device efficiency. As shown in Fig. 6d, the current-density-dependent variations in the current efficiency indicate that under the same current flow, the radiative recombination of the electrically excited QDs is significantly more efficient in the CdSe/ZnS/Al-QD-based QLEDs compared to that in the CdSe/ZnS-QD-based QLED^27^. In our device architecture, the electron injection into the EMLs precedence over the hole injection^28^. The accompanying accumulation of excess electrons reduces the efficiency of individual QDs through Auger recombination^29^, particularly as the excitation density increases. However, the Al_2_O_3_ shells acts as a barrier between the ETL and QD, balancing the injection of electrons and holes. As a result, QDs with Al shells exhibit higher efficiencies and excellent exciton generation rates.

**Table 1:** Device characteristics of all QLEDs.

Device	Turn-on Voltage @ 1 cd/m^2^ (V_on_)	Maximum Luminance (cd/m^2^)	Current Efficiency @ Max luminance (cd/A)	CIE (x, y) @ Max luminance
QLED1	2.0	36,580	3.39	0.30, 0.66
QLED2	1.8	48,650	4.69	0.29, 0.68
QLED3	1.8	57,580	5.48	0.30, 0.67
QLED4	2.2	41,940	4.55	0.28, 0.68

### Lifetime characteristics of the QLEDs

**Figure 7:**
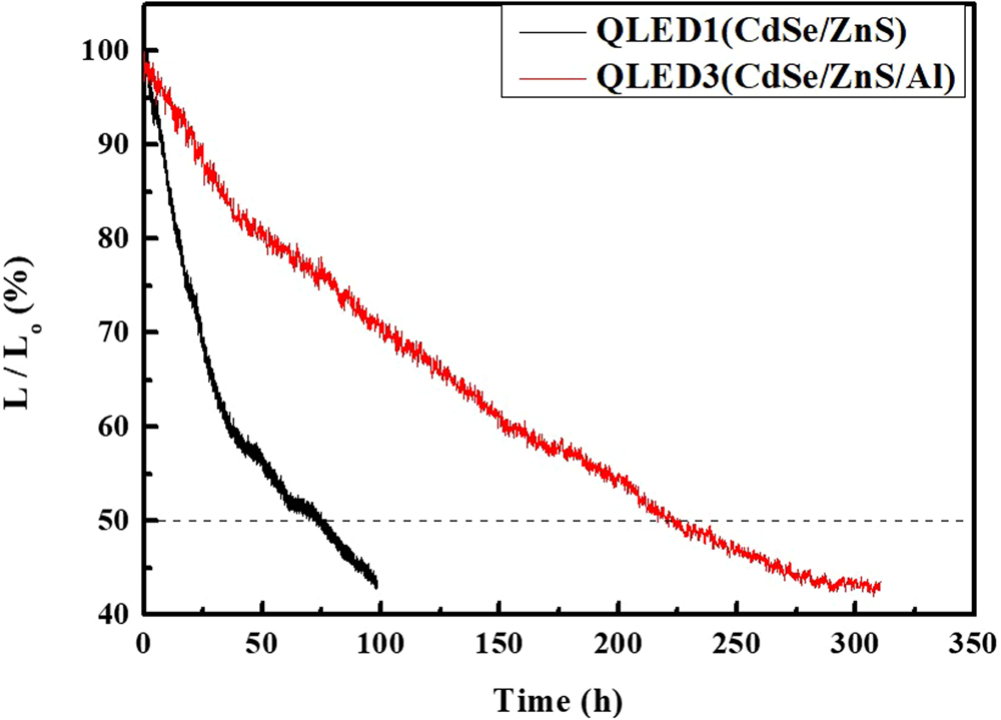
Lifetime characteristics of un-encapsulated QLED1 and QLED3. Lifetime characteristics of QLED1 and QLED3 without encapsulation. Lifetime characteristics of the QLED1 (black line) and QLED3 (red line) at the initial luminance of 1,000 cd/m^2^, under constant current operation 5 mA at room temperature.

In order to validate the superiority in device stability, we evaluated the lifetimes of QLED1 and QLED3. The lifetime characteristics of the unencapsulated QLEDs were assessed by operating the devices at a constant current of 5 mA, as shown in Fig. 7. All of the lifetime characteristics were performed under ambient conditions. The lifetime T50 (measured in hours) is the time required for the luminance to decrease to 50% of its initial value. The luminance of QLED1 deteriorated rapidly from its initial luminance of 1,000 cd/m^2^, reaching T50 after 75.6 h of continuous operation. In contrast, the luminance of QLED3 (initially 1,000 cd/m^2^) slowly decayed, reaching T50 after 226 h. These results clearly show that QLED3 was more stable under the operation conditions and that its lifetime was almost three times longer than that of QLED1. These excellent device-stability of the QLED3 are attributed to the efficient QD passivation owing to the Al_2_O_3_ shell, which serves as a physical barrier to penetration of oxygen.

## Discussion

We developed a simple approach to improve the device stability and photostability of gradient CdSe/ZnS QDs using an outer Al_2_O_3_ shell. In the operating devices based on CdSe/ZnS/Al QDs, the energy loss due to Auger recombination was considerably reduced and potentially completely suppressed at driving currents. The resulting solution-processable QLED exhibited an excellent device performance, with a maximum luminance of 57,580 cd/m^2^, maximum current efficiency of 5.48 cd/A, low turn-on voltage (≤2 V), and operation lifetime of 226 h. The considerable improvements in the photostability and device stability were attributed to the self-passivation characteristics of the Al_2_O_3_ shell, which serves as a physical barrier to penetration of oxygen and moisture.

## Methods

### Synthesis of the CdSe/ZnS and CdSe/ZnS/Al QDs

Green CdSe/ZnS QDs with chemical composition gradients were prepared using a method reported in the literature^19,20,30^. For a typical synthesis, 0.4 mmol of CdO, 4 mmol of Zn(acet)_2_∙2H_2_O, and 5 mL of OA were loaded in a 100-mL three-neck flask and evacuated at 150 °C under vacuum degassing for 30 min. After the vacuum degassing, high-purity argon (Ar) gas was purged. Subsequently, 15 mL of 1-Octadecene (ODE) were added to the three-neck flask, the temperature was increased to 320 °C, and a stock solution containing 0.4 mmol of Se and 3.0 mmol of S in 2.0 mL of trioctylphosphine (TOP) was quickly injected into the reactor at the elevated temperature. The reaction temperature was maintained at 320 °C for 10 min for CdSe/ZnS QD growth. For a typical synthesis of CdSe/ZnS/Al QDs, after the reaction for CdSe/ZnS QDs was completed, we performed the dropwise addition of a mixture of Al(IPA)_3_ dissolved in DDT at 235 °C. This Al shelling was maintained at 235 °C for 2 h. All of the synthesized QDs were purified by adding a toluene and ethanol solution. The mixed solution was centrifuged at 3,000 rpm for 10 min to separate the QDs through precipitation. The supernatant liquid phase was decanted to remove the excess reagent. Subsequently, the QDs were redispersed in a non-polar toluene solution (20 mg/mL).

### Device fabrication and characterization

The QLEDs were fabricated by spin-coating on glass substrates, which were commercially pre-coated with an indium tin oxide (ITO) anode (150 nm). The substrates were cleaned in consecutive ultrasonic baths of acetone, methanol, and deionized water (for 10 min in each of them), and then exposed to UV ozone for 15 min. In order to form the HIL, the substrates were spin-coated with PEDOT:PSS (Baytron P AI 4083), and baked at 150 °C for 10 min in air to form the HIL. The HTL was formed using poly-TPD (SOL2420H, Solaris Chem), which is a good resistor for non-polar organic solvents such as toluene, dissolved in chlorobenzene at 0.5 wt%. After the spin coating, the HTL was baked at 110 °C for 30 min under vacuum conditions. The QDs were spin-coated to form the EML. Annealing was performed for 30 min at 80 °C under vacuum conditions. The ETL was formed by spin-coating a zinc oxide (ZnO) NP ethanol dispersion with a concentration of 30 mg/mL for 30 min at 90 °C under vacuum conditions. The ZnO NPs were synthesized using a modified version of a previously reported method^19,20,31–33^; their sizes were 4–6 nm (Supplementary Fig. S6). Subsequently, the emissive area of the fabricated device was defined to be 9 mm^2^. The characteristics of the QLEDs were measured using a Keithley-2400 source-meter unit and luminance meter (CS-100A).

## Supplementary information

Supplementary Information

## References

[1] Colvin VL, Schlamp MC, Alivisatos AP (1994). Light-Emitting-Diodes Made from Cadmium Selenide Nanocrystals and a Semiconducting Polymer. Nature.

[2] Anikeeva PO, Halpert JE, Bawendi MG, Bulović V (2007). Electroluminescence from a Mixed Red−Green−Blue Colloidal Quantum Dot Monolayer. Nano Lett..

[3] Shirasaki Y, Supran GJ, Bawendi MG, Bulović V (2012). Emergence of Colloidal Quantum-Dot Light-Emitting Technologies. Nat. Photon..

[4] Yang X (2014). Stable, Efficient, and All-Solution-Processed Quantum Dot Light-Emitting Diodes with Double-Sided Metal Oxide Nanoparticle Charge Transport Layers. ACS Appl. Mater. Interfaces.

[5] Konstantatos G (2006). Ultrasensitive Solution-Cast Quantum Dot Photodetectors. Nat. Lett..

[6] Semonin OE (2011). Peak External Photocurrent Quantum Efficiency Exceeding 100% via MEG in a Quantum Dot Solar Cell. Science.

[7] Sark WGJHMV (2001). Photooxidation and Photobleaching of Single CdSe/ZnS Quantum Dots Probed by Room-Temperature Time-Resolved Spectroscopy. J. Phys. Chem. B.

[8] Rowland CE (2014). Thermal Stability of Colloidal InP Nanocrystals: Small Inorganic Ligands Boost High-Temperature Photoluminescence. ACS Nano.

[9] Li L (2009). Highly Luminescent CuInS2/ZnS Core/Shell Nanocrystals: Cadmium-Free Quantum Dots for *In Vivo* Imaging. Chem. Mater..

[10] Yang Y (2015). High-Efficiency Light-Emitting Devices Based on Quantum Dots with Tailored Nanostructures. Nat. Photon..

[11] Bae WK (2009). Highly Efficient Green-Light-Emitting Diodes Based on CdSe@ZnS Quantum Dots with a Chemical-Composition Gradient. Adv. Mater..

[12] Wang Q, Iancu N, Seo DK (2005). Preparation of Large Transparent Silica Monoliths with Embedded Photoluminescent CdSe@ZnS Core/Shell Quantum Dots. Chem. Mater..

[13] Chung YS, Jeon MY, Kim CK (2009). Performance Changes of Surface Coated Red Phosphors with Silica Nanoparticles and Silica Nanocomposites. Ind. Eng. Chem. Res..

[14] Jun S, Lee J, Jang E (2013). Highly Luminescent and Photostable Quantum Dot–Silica Monolith and Its Application to Light-Emitting Diodes. ACS Nano.

[15] Jung HS (2015). Silica-Coated Gradient Alloy Quantum Dots with High Luminescence for Converter Materials in White Light-Emitting Diodes. RSC Adv..

[16] Pietra F (2013). Synthesis of Highly Luminescent Silica-Coated CdSe/CdS Nanorods. Chem. Mater..

[17] Rao P (2015). Highly Stable CuInS_2_@ZnS:Al Core@shell Quantum Dots: The Role of Aluminium Self-Passivation. Chem. Commun..

[18] Kim JH (2016). Enhanced Fluorescent Stability of Copper Indium Sulfide Quantum Dots through Incorporating Aluminum into ZnS Shell. J. Alloys Compd..

[19] Kang BH (2016). Efficient Exciton Generation in Atomic Passivated CdSe/ZnS Quantum Dots Light-Emitting Devices. Sci. Rep..

[20] Lee JS (2017). All-Solution-Processed High-Brightness Hybrid White Quantum-Dot Light-Emitting Devices Utilizing Polymer Modified Quantum Dots. Org. Electron..

[21] Lee KH (2014). Over 40 cd/A Efficient Green Quantum Dot Electroluminescent Device Comprising Uniquely Large-Sized Quantum Dots. ACS Nano.

[22] Li Z (2015). General Method for the Synthesis of Ultrastable Core/shell Quantum Dots by Aluminum Doping. J. Am. Chem. Soc..

[23] Loginova E, Cosandey F, Madey TE (2007). Nanoscopic Nickel Aluminate Spinel (NiAl2O4) Formation During NiAl(111) oxidation. Surf. Sci..

[24] Lu CL (2009). Crystalline Nanotubes of γ-AlOOH and γ-Al_2_O_3_: Hydrothermal Synthesis, Formation Mechanism and Catalytic Performance. Nanotechnology.

[25] Shen H (2015). High-Efficiency, Low Turn-on Voltage Blue-Violet Quantum-Dot-Based Light-Emitting Diodes. Nano Lett..

[26] Lim J (2014). Influence of Shell Thickness on the Performance of Light-Emitting Devices Based on CdSe/Zn_1-X_Cd_X_S Core/Shell Heterostructured Quantum Dots. Adv. Mater..

[27] Kim OS (2016). Efficient Quantum Dots Light-Emitting Devices Using Polyvinyl Pyrrolidone-Capped ZnO Nanoparticles With Enhanced Charge Transport. IEEE Electron Device Lett..

[28] Dai X (2014). Solution-Processed, High-Performance Light-Emitting Diodes Based on Quantum Dots. Nat. Lett..

[29] Bae WK (2013). Controlling the Influence of Auger Recombination on the Performance of Quantum-Dot Light-Emitting Diodes. Nat. Commun..

[30] Bae WK, Char K, Hur H, Lee S (2008). Single-Step Synthesis of Quantum Dots with Chemical Composition Gradients. Chem. Mater..

[31] Meulenkamp EA (1998). Synthesis and Growth of ZnO Nanoparticles. J. Phys. Chem. B..

[32] Asok A, Gandhi MN, Kulkarni AR (2012). Enhanced visible photoluminescence in ZnO quantum dots by promotion of oxygen vacancy formation. Nanoscale.

[33] Pacholski C, Kornowski A, Weller H (2002). Self-Assembly of ZnO: From Nanodots to Nanorods. Angew. Chem. Int. Ed..

