# Supplementary material for: Al atomistic surface modulation on colloidal gradient quantum dots for high-brightness and stable light-emitting devices
Source: Sci Rep. 2019 Apr 23;9:6357. doi: 10.1038/s41598-019-42925-0 (PMC6478915; doi:10.1038/s41598-019-42925-0)
Supplement: Supplementary file 1 — Supplementary Information [file 41598_2019_42925_MOESM1_ESM.pdf]

Supplementary Information

Al atomistic surface modulation on colloidal gradient quantum dot for brightness and stable light-emitting devices

Jae-Sung Lee[1,*], Byoung-Ho Kang[2,*], Sae-Wan Kim[3], Jin-Beom Kwon[3], Ok-Sik Kim[3], Young Tae Byun[1], Dae-Hyuk Kwon[4], Jin-Hyuk Bae[3], and Shin-Won Kang[3,†]

[1]Sensor System Research Center, Korea Institute of Science and Technology (KIST), 5 Hwarang-ro 14-gil, Seongbuk-gu, Seoul 02792, Republic of Korea

[2]Institute of Technology, DONG-A CARBON TECHNOLOGY, 41-3, Gyo 8-Gil, Buksam-eub, Chilgok-gun, Gyeongsangbuk-do, Republic of Korea

[3]School of Electronics Engineering, College of IT Engineering, Kyungpook National University, 1370 Sankyuk-dong, Bukgu, 702-701 Daegu, Republic of Korea

[4]Department of Electronic Engineering, Kyungil University, Hayang-up, 712-702 Gyeongsang buk-do, Republic of Korea

*Corresponding author: Prof. Shin-Won Kang (swkang@knu.ac.kr)

1. Energy gap (E_g) of CdSe/ZnS QDs after Al-passivation.

The energy levels of the electronic states correspond to the energy carried by UV or visible radiation. At resonance, the molecules can absorb quantified energy transported by the electromagnetic radiation, and promote an electron from a low-energy molecular orbital to a higher energy molecular orbital[1,2]. These transitions can be measured using a UV-visible absorption spectrometer. The energy gap (E_g) can be determined by extrapolating the linear region down to zero absorption in the longer wavelength region, and was found to be 2.29 eV using equation (1).

$$E_g(eV) = \frac{1242}{\lambda\,(nm)} \tag{1}$$

The energy gap of the materials, measured from the optical absorption curves, can be related to the absorbance (a), by following equation (2).

$$ahv = A(hv - E_g)^{1/2} \tag{2}$$

where hv is the energy of the photon and A is the proportionality constant.

As shown in Fig. 2a, for the two absorption spectra of CdSe/ZnS and CdSe/ZnS/Al QDs, it is worth nothing that no significant difference in UV-visible absorption spectrum was observed between the QDs with/without Al-passivation. These results indicate that there is no significant difference observed in the energy gap between the QDs with/without Al-passivation.

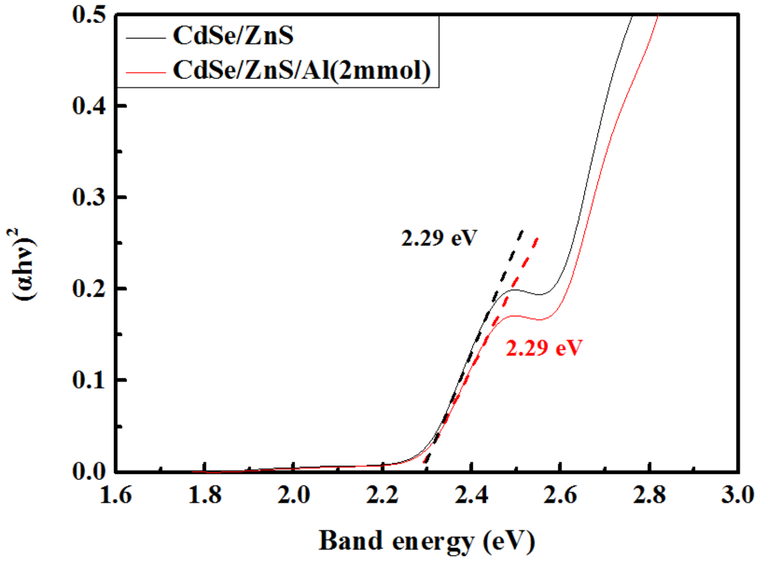

Figure S1. The plots $(ahv)^2$ versus hv relations converted from the absorption spectra of CdSe/ZnS QDs after Al-passivation.

2. Photoluminescence quantum yield (PL QY) of CdSe/ZnS QDs after Al-passivation.

The absolute quantum yield of QD solutions was obtained by the absolute PL QY measurement system (OTSUKA Electronics, QE-2000) excited at 450 nm. The Al-passivated QDs exhibit a higher PL QY than that of the bare QDs, as shown in supplementary information Fig. S2. These results show that Al-passivation of the QDs surface leads to generates a more efficient thin film EML than that achieved bare by QDs.

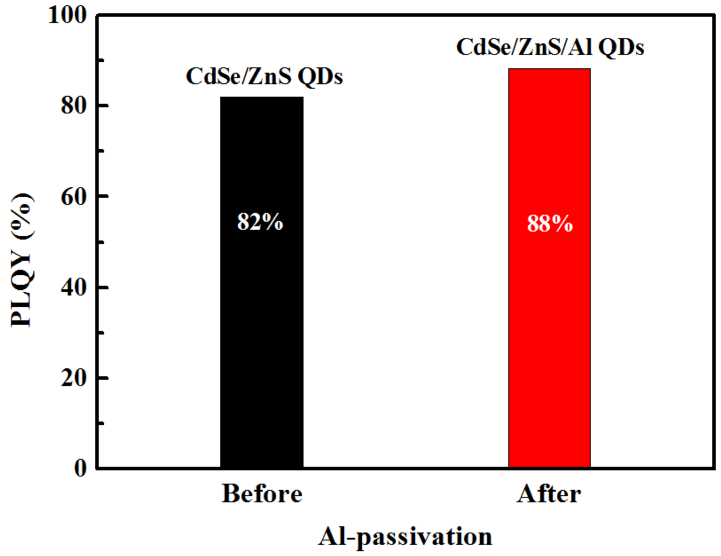

Figure S2. The PL QY of CdSe/ZnS QDs after Al-passivation.

3. Effects of overshelling time on QY of CdSe/ZnS/Al QDs with 2 mmol doping concentration

Figure S3 shows that the reaction time had a significant effect on the QY of the CdSe/ZnS/Al QDs. The highest QY was achieved with a reaction time of 2 h.

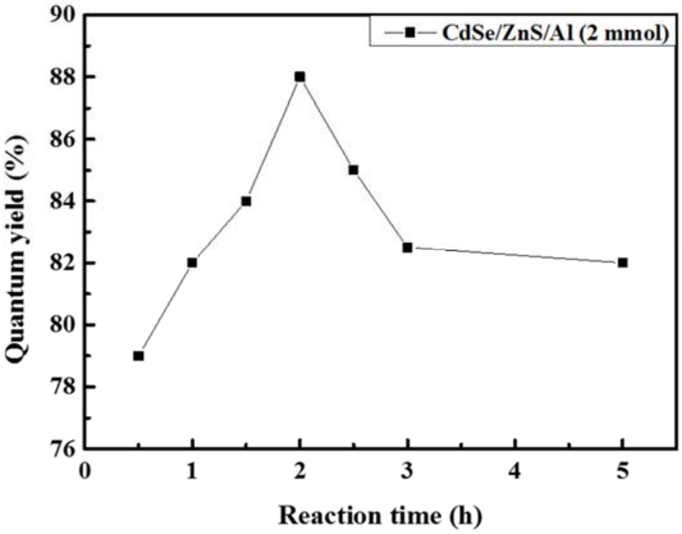

Figure S3. Effects of overshelling time on QY of CdSe/ZnS/Al QDs with 2 mmol doping concentration.

4. Component analysis of CdSe/ZnS and CdSe/ZnS/Al QDs

To verify the passivation of the Al on the CdSe/ZnS QD surfaces, we analyzed the energy dispersive spectroscopy (EDS) results. The atomic percentage of the Al was calculated to be approximately 4.3%, as shown in Figure S4. These results confirm that the Al was well-shelled on the QD surface.

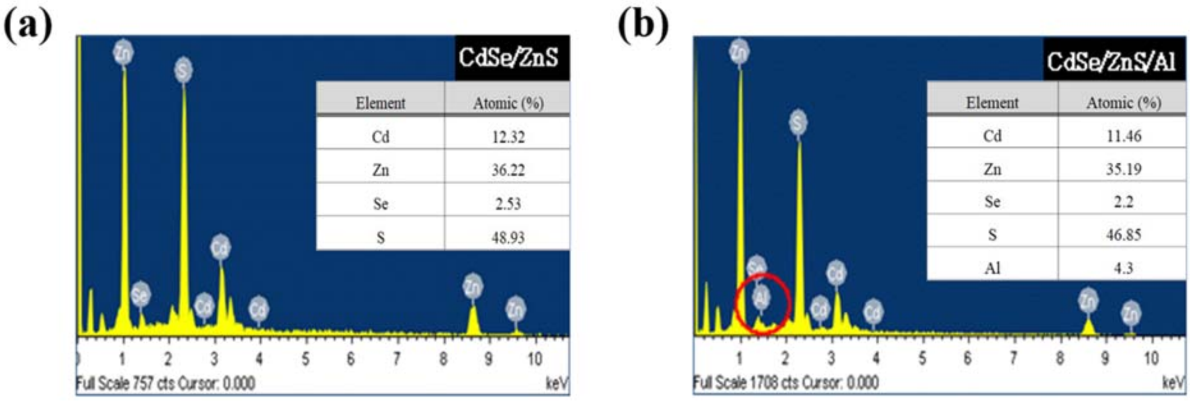

Figure S4. EDS spectra and elemental compositions (insets) of QDs. (a) CdSe/ZnS QDs and (b) CdSe/ZnS/Al QDs.

5. Electrical measurements on the electron-only devices

In order to confirm the contribution the Al shelling of the QDs in promoting electron injection and transport, the current densities of the electron-only devices with and without Al shelling were measured (Supplementary Fig. S5). The current density of electron-only device (ITO/Al/QDs(CdSe/ZnS/Al)/Al) is much larger than that of device (ITO/Al/QDs(CdSe/ZnS)/Al). In the above two devices, the thickness of all layers are identical to those used in the QLEDs. This result clearly demonstrates that the electron injection and transport in the devices with CdSe/ZnS/Al QDs are enhanced by the thicker Al shell acting as a nontrivial energy barrier against the charge injection.

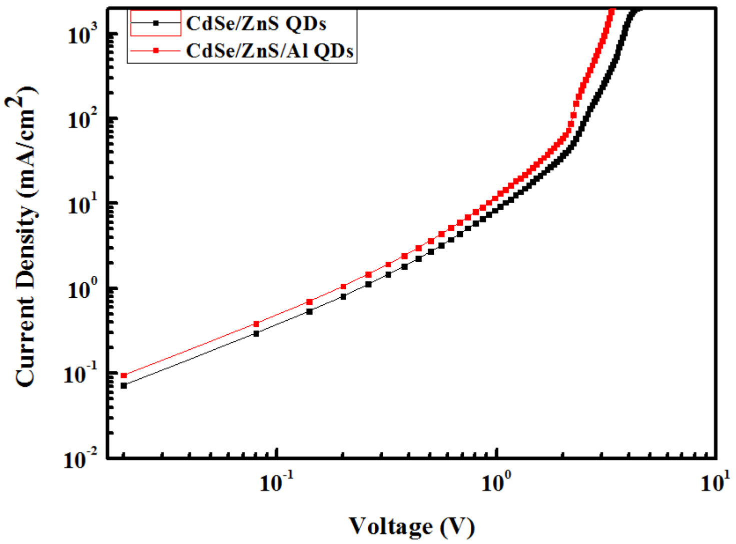

Figure S5. Current density-voltage (J-V) characteristics of electron-only devices with and without Al shelling of the QDs.

6. Synthesis and purification of zinc oxide (ZnO) nanoparticles

Materials. Zinc acetate dehydrate (Zn(acet)$_2$·2H$_2$O, 99%), extra pure ethanol, and anhydrous methanol were purchased from Sigma-Aldrich. Potassium hydroxide (KOH, AR reagent), 2-propanol, and hexane were obtained from Duksan Pharmaceutical Co. Ltd. All chemicals were used as received, without further purification.

Synthesis of ZnO nanoparticles via the sol-gel method. A modification of the sol-gel method was used for the synthesis of ZnO nanoparticles (NPs) in alcohol solution[3-6]. The solutions were prepared using 2.46 g of Zn(acet)$_2$·2H$_2$O and 0.96 g of KOH dispersed in 110 and 50 mL of methanol, respectively. The Zn(acet)$_2$·2H$_2$O solution was placed in a 200 mL flask and heated to 60 °C and the KOH solution was added by droplets (1 mL/s). The mixture was stirred at 60 °C for 60 min and then allowed to cool. To obtain uniform ZnO NPs, we implemented the necessary aging process by adding 2-propanol and hexane overnight. The ZnO NPs were then precipitated via centrifugation at 3,000 rpm and re-dispersed in ethanol (30 mg/mL). To determine the characteristics of the ZnO NPs, we performed UV-visible spectrum analysis and transmission electron microscopy (TEM), as shown in Figure S6.

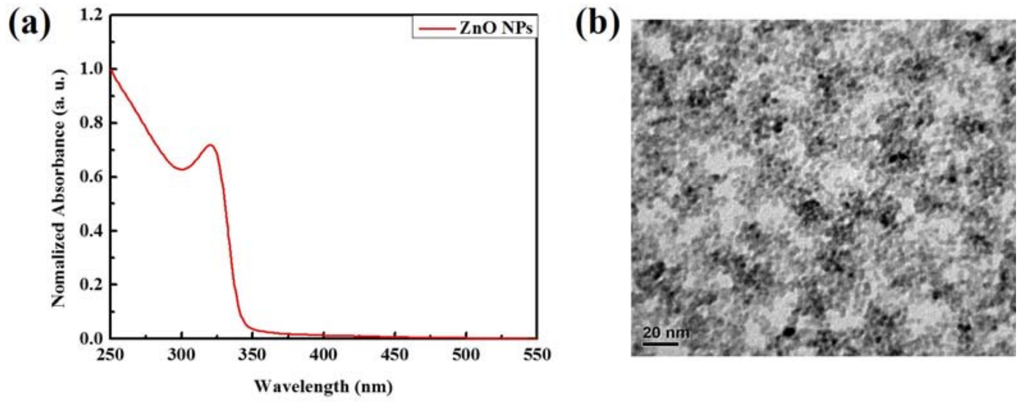

Figure S6. The characteristics of synthesized ZnO NPs. (a) UV-Vis absorption spectrum and (b) TEM image.

7. References

1. Segets, D. et al. Determination of the Quantum Dot Band Gap Dependence on Particle Size from Optical Absorbance and Transmission Electron Microscopy Measurements. *ACS Nano*. **6**, 9021–9032 (2012).

2. Kong, E. H., Chang, Y. J., Park, H. J., & Jang, H. M. Bandgap Tuning by Using a Lattice Distortion Induced by Two Symmetries That Coexist in a Quantum Dot. *Small*. **10**, 1300–1307 (2014).

3. Meulenkamp, E. A. Synthesis and Growth of ZnO Nanoparticles. *J. Phys. Chem. B*. **102**, 5566–5572 (1998).

4. Asok, A.; Gandhi, M. N. & Kulkarni, A. R. Enhanced visible photoluminescence in ZnO quantum dots by promotion of oxygen vacancy formation. *Nanoscale* **4**, 4943-4946 (2012).

5. Pacholski, C.; Kornowski, A.; Weller, H. Self-Assembly of ZnO: From Nanodots to Nanorods. *Angew. Chem. Int. Ed*. **41**, 1188-1191 (2002).

6. Kang, B. H. *et al* Efficient exciton generation in atomic passivated CdSe/ZnS quantum dots light-emitting devices. *Scientific Reports* **6**, 34659 (2016).
